# Estimating the volume of penumbra in rodents using DTI and stack-based ensemble machine learning framework

**DOI:** 10.1186/s41747-024-00455-z

**Published:** 2024-05-15

**Authors:** Duen-Pang Kuo, Yung-Chieh Chen, Yi-Tien Li, Sho-Jen Cheng, Kevin Li-Chun Hsieh, Po-Chih Kuo, Chen-Yin Ou, Cheng-Yu Chen

**Affiliations:** 1 Department of Medical Imaging, Taipei Medical University Hospital, No.250, Wu Hsing Street, Taipei, Taiwan; 2 Translational Imaging Research Center, Taipei Medical University Hospital, Taipei, Taiwan; 3 Research Center for Neuroscience, Taipei Medical University, Taipei, Taiwan; 4 Ph.D. Program in Medical Neuroscience, College of Medical Science and Technology, Taipei Medical University, Taipei, Taiwan; 5 Department of Computer Science, National Tsing Hua University, Hsinchu, Taiwan; 6 Research Center for Artificial Intelligence in Medicine, Taipei Medical University, Taipei, Taiwan; 7 Department of Radiology, School of Medicine, College of Medicine, Taipei Medical University, Taipei, Taiwan; 8 Department of Radiology, National Defense Medical Center, Taipei, Taiwan

**Keywords:** Animals, Diffusion tensor imaging, Infarction (middle cerebral artery), Ischemic stroke, Machine learning

## Abstract

**Background:**

This study investigates the potential of diffusion tensor imaging (DTI) in identifying penumbral volume (PV) compared to the standard gadolinium-required perfusion–diffusion mismatch (PDM), utilizing a stack-based ensemble machine learning (ML) approach with enhanced explainability.

**Methods:**

Sixteen male rats were subjected to middle cerebral artery occlusion. The penumbra was identified using PDM at 30 and 90 min after occlusion. We used 11 DTI-derived metrics and 14 distance-based features to train five voxel-wise ML models. The model predictions were integrated using stack-based ensemble techniques. ML-estimated and PDM-defined PVs were compared to evaluate model performance through volume similarity assessment, the Pearson correlation analysis, and Bland–Altman analysis. Feature importance was determined for explainability.

**Results:**

In the test rats, the ML-estimated median PV was 106.4 mL (interquartile range 44.6–157.3 mL), whereas the PDM-defined median PV was 102.0 mL (52.1–144.9 mL). These PVs had a volume similarity of 0.88 (0.79–0.96), a Pearson correlation coefficient of 0.93 (*p* < 0.001), and a Bland–Altman bias of 2.5 mL (2.4% of the mean PDM-defined PV), with 95% limits of agreement ranging from -44.9 to 49.9 mL. Among the features used for PV prediction, the mean diffusivity was the most important feature.

**Conclusions:**

Our study confirmed that PV can be estimated using DTI metrics with a stack-based ensemble ML approach, yielding results comparable to the volume defined by the standard PDM. The model explainability enhanced its clinical relevance. Human studies are warranted to validate our findings.

**Relevance statement:**

The proposed DTI-based ML model can estimate PV without the need for contrast agent administration, offering a valuable option for patients with kidney dysfunction. It also can serve as an alternative if perfusion map interpretation fails in the clinical setting.

**Key points:**

• Penumbral volume can be estimated by DTI combined with stack-based ensemble ML.

• Mean diffusivity was the most important feature used for predicting penumbral volume.

• The proposed approach can be beneficial for patients with kidney dysfunction.

**Graphical Abstract:**

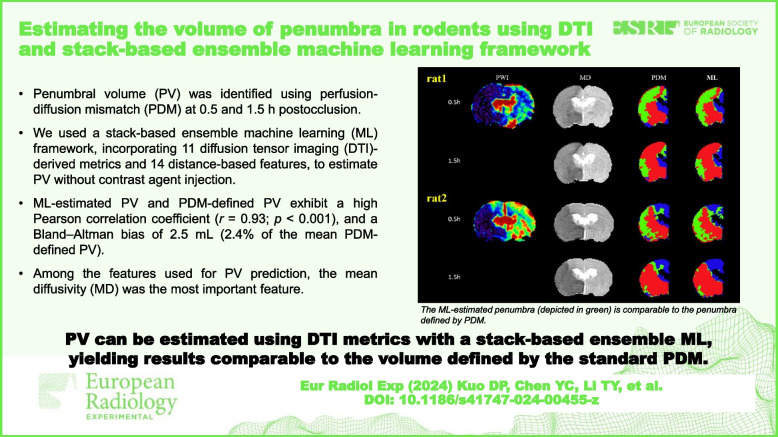

**Supplementary Information:**

The online version contains supplementary material available at 10.1186/s41747-024-00455-z.

## Background

Stroke is a leading cause of disability and mortality worldwide. Approximately 80% of all cases are attributed to ischemic stroke. In the management of patients with acute ischemic stroke (AIS), magnetic resonance imaging (MRI) plays key roles in both diagnosis and treatment planning [1]. The integration of diffusion-weighted imaging (DWI) and perfusion-weighted imaging (PWI) enables the identification of potentially salvageable penumbra through the concept of perfusion–diffusion mismatch (PDM), facilitating the assessment of patients’ eligibility for mechanical thrombectomy [2]. However, technical complexities associated with PWI, limitations related to intravenous access, and contraindications to contrast agents reduce the clinical application of PWI [3, 4].

Recent emphasis on DWI has led to the inclusion of diffusion tensor imaging (DTI) in routine brain MRI protocols. In clinical settings, DTI has been used to identify neurological disorder-induced changes in cerebral microstructures [5]. Compared with standard DWI, DTI coupled with a parallel acquisition technique generates higher-quality trace images and exhibits enhanced sensitivity for detecting small cerebral infarctions [6]. DTI-derived metrics can help assess ischemic brain tissue damage [7], determine AIS onset time in both animals [8] and humans [9], estimate the salvageable tissue [8], and differentiate between benign cerebral blood flow reduction and the penumbral tissue on the basis of microstructures [10]. Therefore, DTI can provide comprehensive insights into the pathophysiological process of cerebral ischemia.

Machine learning (ML) has emerged as a valuable tool in the medical field. Numerous ML algorithms have been developed, and selecting the most effective predictive algorithm for a specific task is crucial. Stacked generalization [11], commonly referred to as “stacking,” is an ensemble method that is extensively used in various domains to address the challenge of selecting the most appropriate algorithm and achieving superior performance compared with that of a single algorithm [12]. However, this stack-based ensemble technique often places considerable emphasis on accuracy while overlooking the model interpretability [13].

In this experimental study, we leveraged the advantages of the DTI and stacking techniques for penumbra imaging. We hypothesized that a stack-based ML model would provide accurate and reliable estimates of the penumbral volume (PV) while simultaneously improving the explainability of penumbra segmentation.

## Methods

### Animals

All animal experiments were ethically approved by the Institutional Animal Care and Use Committee of Taipei Medical University (approval No: LAC-2022–0069). Twenty-five male Sprague Dawley rats (weight: 250–300 g) were used. The rats were housed in a controlled environment with maintained humidity and temperature. They were subjected to a 12-h light/dark cycle and provided ad libitum access to sterile food and water. Permanent middle cerebral artery occlusion was induced in all rats by using a previously reported intraluminal suture method [14].

In brief, rats were anesthetized with chloral hydrate (450 mg/kg; Sigma, St. Louis, MO, USA) in the supine position. An incision below the mandible exposed the left common carotid artery and its branches—the internal carotid artery and the external carotid artery. A 3–0 surgical nylon suture with a heat-rounded tip (length 50 cm; UNIK Surgical Sutures, Taiwan) was inserted into the opening of the external carotid artery and then into the internal carotid artery. After the removal of silk sutures from the common carotid artery and internal carotid artery, the nylon suture was advanced into the internal carotid artery until resistance was encountered. The incision was sutured, and the rat was prepared for MRI. Five rats that exhibited ischemic core (IC) regions not involving the cerebral cortex and four rats that died during image acquisition were excluded from this study. Thus, 16 rats were included in the final analysis.

### Image acquisition

Images were acquired using a 7-T scanner (PharmaScan 70/16; Bruker, Ettlingen, Germany). The rats were anesthetized using 1.5–2% isoflurane and their rectal temperature was maintained at approximately 37 °C by placing them in a warm water bath with continuous circulation; the temperature was controlled by an external controller. DTI was performed using 30 noncollinear diffusion-encoding gradient directions with a *b* factor of 1,200 s/mm^2^ and five *b* = 0 s/mm^2^ measurements. Multishot echo-planar imaging was performed with the following technical parameters: repetition time 3,000 ms, echo time 37 ms; number of excitations 2; number of slices 16; section thickness 1 mm, without interslice gap. The navigator-echo correction technique was used as the signal readout module. To detect the presence of penumbra, DTI was performed at 0.5 and 1.5 h after middle cerebral artery occlusion, before the development of the final infarct [15]. PWI was performed at 0.5 h after occlusion by using a dynamic susceptibility contrast technique. A series of gradient-echo echo-planar coronal images were obtained (repetition time 600 ms, echo time 20 ms, repetitions 200). A 0.25 mmol/kg bolus of the susceptibility contrast agent gadobutrol (Gadovist, Bayer Healthcare, Berlin, Germany) was manually injected through the rat tail vein approximately 30 s after the initiation of image acquisition. The images acquired through DTI and PWI were reconstructed using a field of view of 25.6 × 25.6 mm^2^ and a matrix of 96 × 96 and then zero-filled to a matrix of 128 × 128 with a resolution of 0.2 × 0.2 mm^2^ for further analyses.

### PWI and DTI metrics

The PWI maps and DTI metrics were computed using custom algorithms developed in MATLAB (R2022a release, MathWorks, Inc., Natick, MA, USA) and FMRIB Software Library [16], respectively. Initially, we determined the relative cerebral blood volume and relative mean transit time by using the integral and normalized first moment of gamma variate fitting, respectively. Subsequently, the relative cerebral blood flow was derived as the ratio of relative cerebral blood volume to relative mean transit time by using the central volume principle [17]. For the DTI metrics, we computed the eigenvalues of each voxel’s image and combined them to obtain 11 metrics, which were categorized into three classes: *anisotropies* (fractional anisotropy [FA] and relative anisotropy), *diffusivities* (pure isotropic diffusion [p], pure anisotropic diffusion [q], mean diffusivity [MD], radial diffusivity, and axial diffusivity), and *tensors* (covering the total magnitude of diffusion tensor [L], linear tensor, planar tensor, and spherical tensor) [18]. The MD map was subjected to Otsu thresholding [19] for the segmentation of the tissue into the cerebrospinal fluid space and brain parenchyma; for this, the threshold of 800 × 10^-6^ mm^2^/s [20]. The cerebrospinal fluid space was excluded from the other 10 maps on the basis of the results of the cerebrospinal fluid-excluded MD map.

### Delineation of the penumbra, IC and normal tissue

The labels corresponding to the penumbra, IC, and normal tissue (NT) regions were established in advance for supervised learning. Initially, the templates of MD and the midline of the rat’s brain were generated using presurgical data from the rats. Subsequently, the MD template was aligned on the MD map of each rat to create each rat’s midline, which facilitated the automatic separation of the brain into the ipsilateral and contralateral hemispheres [21]. Based on a previous study [8], we defined abnormal MD (*i.e*., the IC) as a 30% reduction in the contralateral hemisphere, excluding the ventricles. Perfusion deficit was defined as a reduced cerebral blood flow (CBF), set at a 46% reduction in the contralateral hemisphere. The CBF and MD maps were coregistered to delineate the penumbra region. Regions in the ipsilateral hemisphere that exhibited no CBF deficits were designated as NT. Contiguity correction was performed to remove “misclassified” voxels [22]. The regions corresponding to the penumbra, IC, and NT were depicted, and the respective voxels were labeled. PV was calculated as the sum of penumbral regions across each slice and multiplied by the slice thickness.

### Machine learning methods

#### Feature extraction

Five types of features (DTI-derived metrics, Mahalanobis, cosine, correlation, and standardized Euclidean distances) were extracted from the regions of interest for each voxel. DTI-derived metrics: 11 DTI-derived metrics were computed for each voxel within the non-IC (penumbra and NT) and IC regions. Once the non-IC and IC matrices were prepared (11 multivariate measurements for an observation [voxel]), four types of distance-based features were conducted. Mahalanobis, cosine, and correlation distances: These distance-based features for a voxel in the non-IC region were computed using the IC region as a reference. The Mahalanobis distance represents the distance of a point from the center of a distribution [23]. Cosine and correlation distances were used to assess similarities between two observations [24], with values closer to 1 indicating greater similarity and those closer to -1 indicating greater dissimilarity. The Mahalanobis distance of each observation in the non-IC matrix was computed relative to the reference observations in the IC matrix. For the cosine/correlation distance, the mean cosine/correlation distance for a voxel was obtained by averaging all cosine/correlation distances between each pair of observations in the non-IC and IC matrices. Three distance-based features were prepared for each voxel. Standardized Euclidean distances: because of the varying units of DTI-derived metrics, the standardized Euclidean distance was calculated separately for each DTI-derived metric, which resulted in 11 features for each voxel. Thus, each voxel was characterized using 25 features, including 11 DTI-derived metrics, 1 Mahalanobis distance, 1 cosine distance, 1 correlation distance, and 11 standardized Euclidean distances as well as their corresponding label.

#### Feature selection

The neighborhood component analysis (NCA) algorithm [25] was used for feature selection to address overfitting and remove potentially redundant features. The regularization parameter *λ* was introduced in the NCA algorithm, and its value was tuned to minimize classification loss [26]. The optimal value of *λ* (*λ*_best_) corresponding to the minimum average classification loss was selected. Using *λ*_best_, the NCA was run on the training data to evaluate the weights of each feature. Features with weights exceeding 2% of the maximum feature weight were selected [25] (Supplementary Fig. S1). Feature selection becomes unnecessary if the value of the generalization error after fitting the NCA model is larger than that obtained before model fitting.

#### Stack-based ensemble learning

Stack-based ensemble learning was used to combine several heterogeneous base models by using outputs from these models to train a final model (*i.e*., stacking model) with improved performance [12]. Five base models (multilayer perceptron (MLP) [27], generalized additive model (GAM) [28], decision tree [29], random forest (RF) [30], and boosting [30]) were individually trained. Subsequently, a final stacking model was constructed by integrating the predictions from the five trained base models along with their respective optimal hyperparameters.

#### Training

**Fig. 1:**
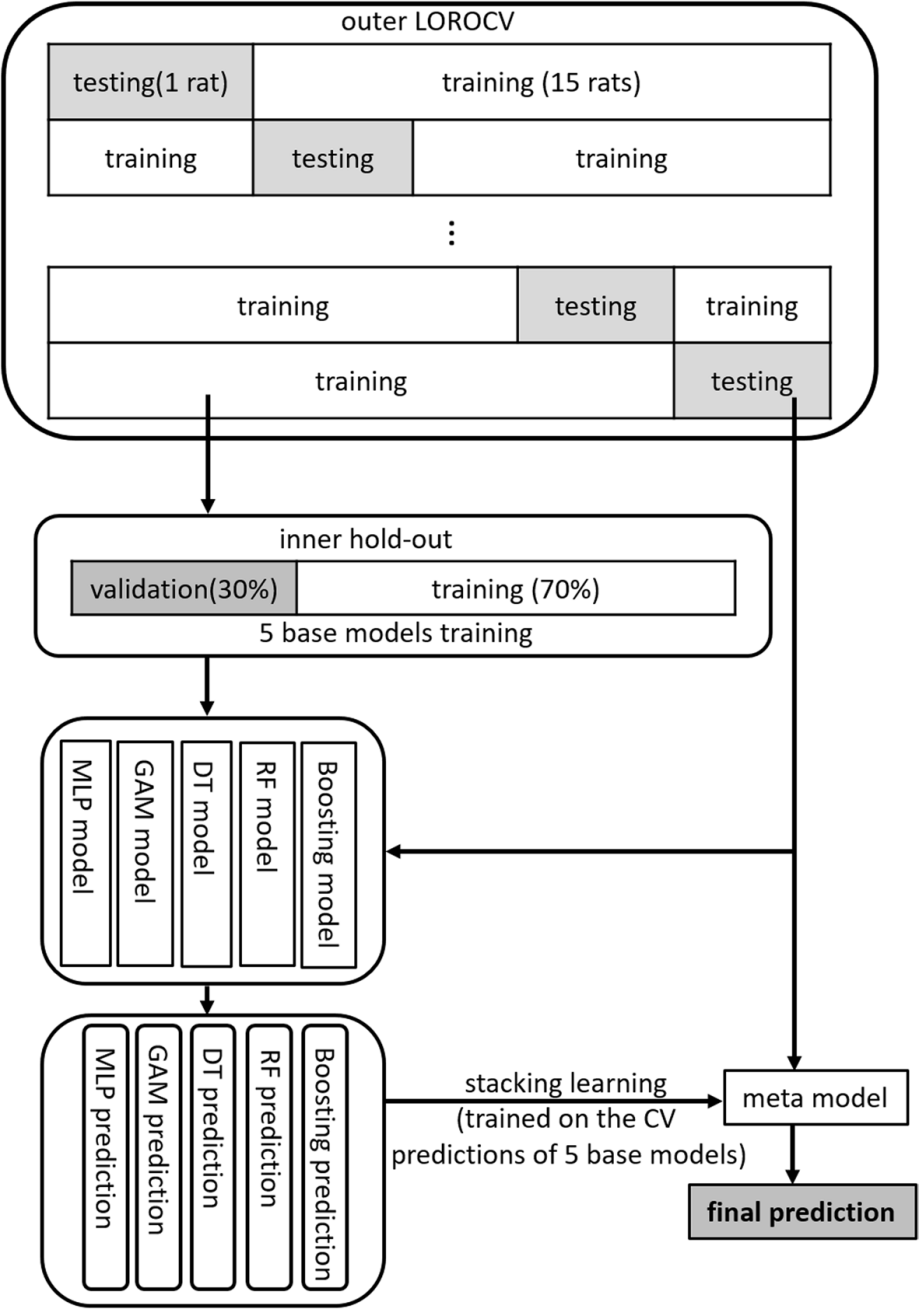
Study framework. *LOROCV* Leave-one-rat-out cross-validation, *MLP* Multilayer perceptron, *GAM* Generalized additive model, *DT* Decision tree, *RF* Random forest

In the training step, a leave-one-rat-out cross-validation scheme outer with nested hold-out inner iterations was implemented (Fig. 1). At each iteration, the data were divided into a training set (15 rats) and a test set (remaining one rat). In addition, the training set was split into an inner training set and a validation set by using the hold-out method (hold-out ratio: 0.3) to fine-tune the model hyperparameters through the Bayesian approach. To address the imbalance between the penumbra and NT classes (penumbra, NT voxels: 39 to 61%), the voxels of the NT class were randomly downsampled, ensuring a 1:1 ratio of penumbra voxels to NT voxels for each rat. Finally, a total of 146,840 class-balanced voxels were obtained from the 16 rats; six models (five base and one stacking) were trained using MATLAB’s Machine-Learning Toolbox and Statistics Toolbox.

#### Feature importance analysis

The permutation technique was used to estimate the importance of each feature. Feature importance permutation was performed for the 16 rats by using the leave-one-rat-out cross-validation method for the RF algorithm [31]. Then, the values of the top six features were calculated from the penumbra and NT classes.

### Validation and statistical analysis

The performance of the six models was evaluated using various indicators, including accuracy, sensitivity, specificity, precision (also known as positive predictive value), F1-score, and area under the receiver operating characteristic curve (AUROC). McNemar test was conducted to determine whether the performance of the stacking model was significantly superior to that of the individual base models during the testing step. Dice similarity coefficient (DSC) and volume similarity [32] were used to evaluate penumbral segmentation for the stacking model. In addition, rat-to-rat Pearson correlation analysis between the ML-estimated PV and PDM-defined PV was performed to assess the applicability and potential of the proposed approach. Bland–Altman analysis was performed to graphically illustrate the differences between the two measurements, and paired Student’s *t* test was performed to compare the values of the top six features between the penumbra and NT. Data are presented in terms of the median and interquartile range (IQR) values unless indicated otherwise. A *p*-value of < 0.05 was considered to be statistically significant.

## Results

**Fig. 2:**
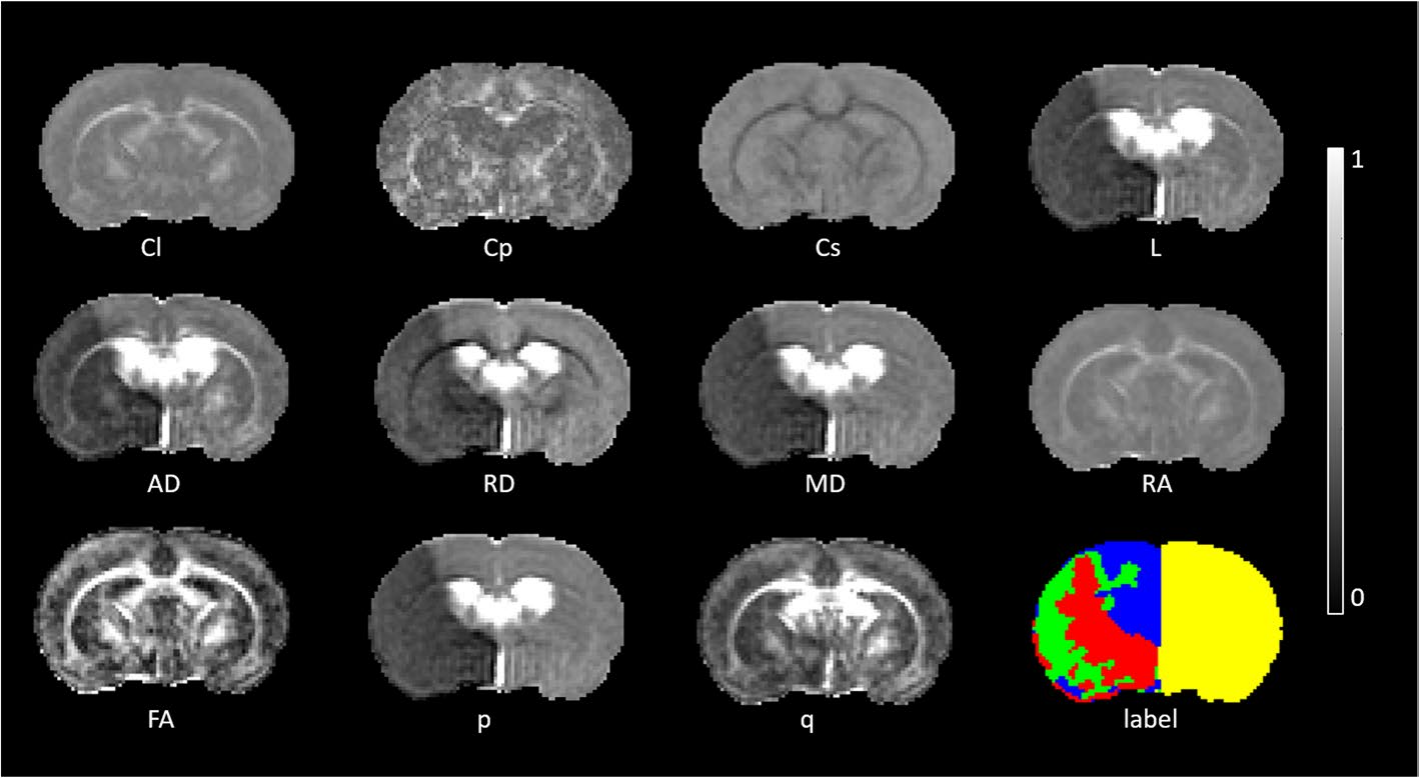
Eleven DTI-derived maps and the corresponding labels for a rat at 0.5 h after MCAO. Each of the 11 DTI-derived maps is displayed along with the corresponding label for a rat at 0.5 h after MCAO. All maps have been normalized to the same grayscale for visual consistency. In the label, red indicates the IC, green indicates the penumbra, blue indicates the NT region, and yellow indicates the contralateral hemisphere. *AD* Axial diffusivity, *Cl* Linear tensor, *Cp* Planar tensor, *Cs* Spherical tensor, *DTI* Diffusion tensor imaging, *FA* Fractional anisotropy, *IC* Ischemic core, *L* Total magnitude of diffusion tensor, *MCAO* Middle cerebral artery occlusion, *MD* Mean diffusivity, *NT* Normal tissue, RA Relative anisotropy, *RD* Radial diffusivity, *p* Pure isotropic diffusion, *q* Pure anisotropic diffusion

In Fig. 2, we present the maps of DTI metrics acquired at 0.5 h after middle cerebral artery occlusion. The maps of L, axial diffusivity, radial diffusivity, MD, and p exhibited initial hypointensity changes in the ischemic areas, whereas the maps of the remaining metrics displayed symmetrical signal intensity. Figure 2 presents the labels for the penumbra, IC, NT, and contralateral hemisphere. The NCA indicated that 25 features would yield the minimum generalization error (Supplementary Table S1). Thus, all 25 features were used for subsequent modeling.

Regarding training performance, the RF model demonstrated the strongest ability to differentiate between the penumbra and the NT, achieving a median accuracy of 0.83 (IQR 0.78–0.87), sensitivity of 0.82 (0.76–0.86), specificity of 0.83 (0.80–0.87), precision of 0.82 (0.77–0.87), F1-score of 0.92 (0.84–0.95), and AUROC of 0.94 (0.83–0.96). The training performances of the other four base models are presented in Table 1.

**Table 1:** Training performance of the five base models

Model	Accuracy	Sensitivity	Specificity	Precision	F1-score	AUROC
MLP	0.75 (0.74, 0.75)	0.72 (0.70, 0.75)	0.77 (0.76, 0.78)	0.75 (0.75, 0.76)	0.74 (0.73, 0.75)	0.80 (0.78, 0.82)
GAM	0.77 (0.60, 0.81)	0.79 (0.49, 0.88)	0.74 (0.73, 0.80)	0.79 (0.74, 0.81)	0.80 (0.56, 0.83)	0.80 (0.68, 0.83)
DT	0.75 (0.75, 0.75)	0.74 (0.73, 0.74)	0.76 (0.76, 0.77)	0.76 (0.75, 0.76)	0.75 (0.74, 0.75)	0.79 (0.79, 0.80)
RF	0.83 (0.78, 0.87)	0.82 (0.76, 0.86)	0.83 (0.80, 0.87)	0.82 (0.77, 0.87)	0.92 (0.84, 0.95)	0.94 (0.83, 0.96)
Boosting	0.76 (0.76, 0.76)	0.76 (0.75, 0.76)	0.77 (0.76, 0.77)	0.76 (0.76, 0.77)	0.76 (0.76, 0.76)	0.84 (0.84, 0.84)

Data are presented as median and interquartile range value

*AUROC* Area under the receiver operating characteristic curve, *MLP* Multilayer perceptron, *GAM* Generalized additive mode, *DT* Decision tree, *RF* Random forest

The test performance of the five trained base models is presented in Table 2. The predictions generated by the five models were used to train the stacking model, which was then applied to the test rats. Regarding test performance, the stacking model exhibited a median accuracy of 0.72 (IQR 0.63–0.80), sensitivity of 0.70 (0.39–0.83), specificity of 0.78 (0.83–0.86), precision of 0.73 (0.65–0.78), F1-score of 0.70 (0.49–0.80), and AUROC of 0.76 (0.66–0.82).

**Table 2:** Test performance of the five base and stacking models

Model		Accuracy	Sensitivity	Specificity	Precision	F1-score	AUROC
	0.5 h	0.73 (0.60, 0.80)	0.80 (0.51, 0.85)	0.75 (0.72, 0.80)	0.78 (0.72, 0.82)	0.78 (0.57, 0.82)	0.78 (0.65, 0.83)
MLP	1.5 h	0.68 (0.62, 0.75)	0.57(0.35, 0.78)	0.81 (0.77, 0.87)	0.67 (0.62, 0.73)	0.52 (0.44, 0.75)	0.69 (0.66, 0.77)
	Overall	0.71 (0.61, 0.79)	0.70 (0.37, 0.83)	0.79 (0.73, 0.86)	0.73 (0.64, 0.78)	0.71 (0.47, 0.79)	0.76 (0.66, 0.82)
	0.5 h	0.77 (0.60, 0.81)	0.79 (0.49, 0.88)	0.74 (0.73, 0.80)	0.79 (0.74, 0.81)	0.80 (0.56, 0.83)	0.80 (0.68, 0.83)
GAM	1.5 h	0.68 (0.64, 0.76)	0.57 (0.34, 0.76)	0.82 (0.77, 0.89)	0.66 (0.63, 0.72)	0.58 (0.43, 0.73)	0.69 (0.64, 0.80)
	Overall	0.71 (0.62, 0.80)	0.69 (0.36, 0.84)	0.78 (0.74, 0.85)	0.73 (0.65, 0.79)	0.68 (0.48, 0.81)	0.76 (0.66, 0.83)
	0.5 h	0.72 (0.62, 0.79)	0.78 (0.51, 0.88)	0.74 (0.70, 0.81)	0.77 (0.72, 0.82)	0.77 (0.58, 0.82)	0.80 (0.69, 0.83)
DT	1.5 h	0.69 (0.64, 0.74)	0.55 (0.34, 0.77)	0.82 (0.74, 0.87)	0.65 (0.62, 0.70)	0.55 (0.44, 0.74)	0.71 (0.65, 0.79)
	Overall	0.70 (0.62, 0.79)	0.70 (0.36, 0.84)	0.77 (0.71, 0.85)	0.71 (0.66, 0.77)	0.69 (0.48, 0.78)	0.75 (0.66, 0.82)
	0.5 h	0.72 (0.60, 0.79)	0.75 (0.47, 0.85)	0.76 (0.71, 0.82)	0.78 (0.70, 0.83)	0.73 (0.55, 0.82)	0.77 (0.66, 0.82)
RF	1.5 h	0.67 (0.65, 0.74)	0.54 (0.32, 0.74)	0.83 (0.77, 0.89)	0.67 (0.62, 0.72)	0.55 (0.42, 0.72)	0.71 (0.64, 0.78)
	Overall	0.74 (0.68, 0.79)	0.75 (0.41, 0.86)	0.77 (0.68, 0.85)	0.72 (0.67, 0.78)	0.73 (0.52, 0.80)	0.77 (0.69, 0.83)
	0.5 h	0.78 (0.68, 0.79)	0.82 (0.60, 0.87)	0.72 (0.66, 0.78)	0.77 (0.71, 0.81)	0.81 (0.65, 0.83)	0.79 (0.72, 0.82)
Boosting	1.5 h	0.72 (0.63, 0.77)	0.77 (0.47, 0.81)	0.79 (0.67, 0.84)	0.68 (0.64, 0.69)	0.71 (0.50, 0.74)	0.74 (0.65, 0.81)
	Overall	0.70 (0.60, 0.79)	0.68 (0.32, 0.81)	0.79 (0.72, 0.85)	0.71 (0.66, 0.78)	0.68 (0.45, 0.78)	0.74 (0.64, 0.82)
	0.5 h	0.75 (0.60, 0.81)	0.81 (0.48, 0.87)	0.75 (0.70, 0.79)	0.78 (0.73, 0.82)	0.79 (0.56, 0.84)	0.80 (0.70, 0.83)
Stacking	1.5 h	0.70 (0.66, 0.77)	0.57 (0.35, 0.76)	0.82 (0.78, 0.88)	0.67 (0.63, 0.72)	0.58 (0.46, 0.75)	0.71 (0.66, 0.79)
	Overall	0.72 (0.63, 0.80)	0.70 (0.39, 0.83)	0.78 (0.73, 0.86)	0.73 (0.65, 0.78)	0.70 (0.49, 0.80)	0.76 (0.66, 0.82)

Data are presented as median and interquartile range value

*AUROC* Area under the receiver operating characteristic curve, *MLP* Multilayer perceptron, *GAM* Generalized additive model, *DT* Decision tree, *RF* Random forest

To analyze the statistical differences between the five base and stacking models, McNemar test was used to compare each base model with the stacking model. Table 3 lists the *p-*values obtained for comparison. In addition, the table also presents the frequency of significant performance improvements observed in the stacking model across 32 tests. As shown in Table 3, the stacking model significantly outperformed the decision tree model in 22 tests but the MLP and GAM models in only 7 and 8 tests, respectively; therefore, the stacking model may not guarantee improvement in all cases. Given their low computational demands, the MLP and GAM models should be preferred over the stacking model.

**Table 3:** Comparison of the stacking model with each base model (*p*-values)

Rat	Time (h)	MLP	GAM	DT	RF	Boosting
Rat1	0.5	0.113	0.898	**0.047**	**0.002**	**0.003**
	1.5	0.550	0.472	**0.018**	**0.000**	**0.001**
Rat2	0.5	0.051	**0.000**	**0.028**	0.324	0.175
	1.5	0.433	**0.000**	**0.000**	**0.000**	**0.000**
Rat3	0.5	0.231	0.765	**0.000**	**0.000**	**0.000**
	1.5	0.531	0.216	**0.031**	**0.007**	**0.000**
Rat4	0.5	1.000	1.000	**0.000**	**0.000**	**0.000**
	1.5	0.339	0.936	**0.003**	0.717	0.087
Rat5	0.5	0.991	0.207	1.000	0.097	0.217
	1.5	**0.001**	0.086	0.100	**0.009**	0.369
Rat6	0.5	**0.000**	0.995	**0.001**	**0.002**	**0.000**
	1.5	**0.000**	0.911	**0.000**	**0.000**	**0.000**
Rat7	0.5	0.742	0.081	**0.000**	**0.029**	**0.001**
	1.5	0.627	**0.000**	**0.000**	**0.000**	**0.000**
Rat8	0.5	0.680	0.135	0.164	0.902	**0.000**
	1.5	1.000	1.000	**0.002**	1.000	**0.000**
Rat9	0.5	**0.000**	0.983	**0.003**	**0.000**	**0.000**
	1.5	**0.004**	**0.003**	**0.001**	**0.000**	**0.000**
Rat10	0.5	0.769	**0.000**	**0.000**	**0.000**	**0.000**
	1.5	0.875	**0.000**	0.069	**0.000**	0.500
Rat11	0.5	**0.002**	0.748	**0.001**	**0.010**	0.114
	1.5	0.948	0.116	**0.002**	0.746	**0.000**
Rat12	0.5	0.656	0.052	0.988	**0.001**	0.343
	1.5	0.215	0.894	**0.019**	**0.025**	0.936
Rat13	0.5	**0.000**	0.982	0.872	0.787	0.999
	1.5	0.198	0.889	**0.036**	0.998	0.664
Rat14	0.5	0.089	1.000	0.928	0.985	0.988
	1.5	0.087	**0.001**	**0.000**	**0.000**	**0.035**
Rat15	0.5	0.594	**0.043**	**0.000**	**0.011**	**0.000**
	1.5	0.156	0.192	0.288	0.526	**0.000**
Rat16	0.5	0.400	0.999	0.882	0.708	1.000
	1.5	0.355	0.583	0.952	0.537	0.348
Frequency for a *p*-value < 0.05	−	7	8	22	20	19

Results in bold indicate significantly superior performance of the stacking model compared with that of the base model

*MLP* Multilayer perceptron, *GAM* Generalized additive model, *DT* Decision tree, *RF* Random forest

**Fig. 3:**
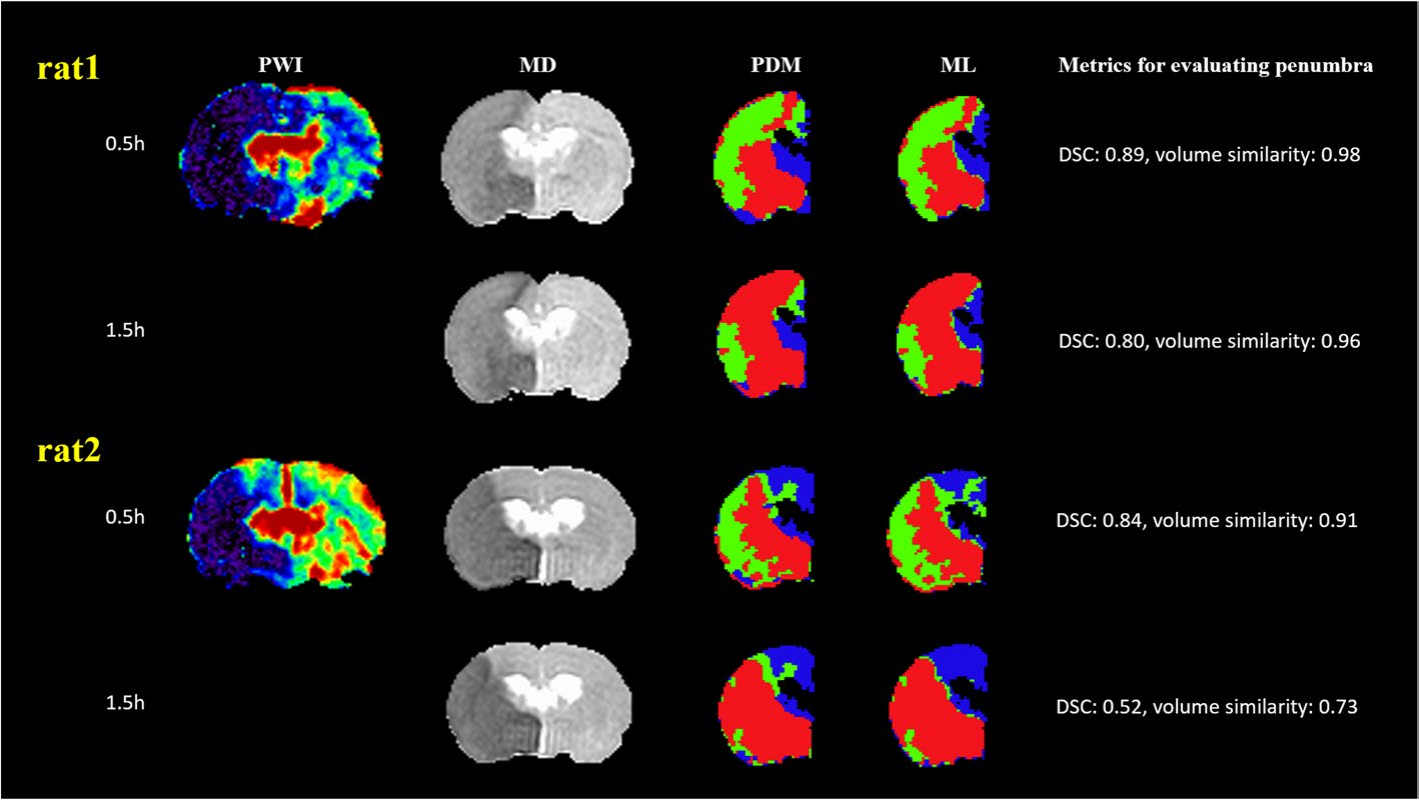
Application of the trained stacking model to two rats. In this study, PWI and DTI were acquired once and twice, respectively. Subsequently, PDM maps at 0.5 and 1.5 h after stroke onset were generated by registering mean MD maps at 0.5 and 1.5 h to PWI, respectively. IC regions are represented in red, penumbra regions in green, and NT regions in blue. The stacking model was trained using data from 15 rats and then applied to the remaining rat. *DSC* Dice similarity score, *DTI* Diffusion tensor imaging, *IC* Ischemic core, *MD* Mean diffusivity, *ML* Machine learning, *NT* Normal tissue, *PDM* Perfusion–diffusion mismatch, *PWI* Perfusion–weighted imaging

Table 4 presents the performance of the stacking model in penumbral segmentation for the test rats. The median ML-estimated PV was 106.4 (IQR 44.6–157.3) mL, whereas the median PDM-defined PV was 102.0 (52.1–144.9) mL. The overall metrics for evaluating penumbra segmentation revealed a median DSC of 0.61 (0.40–0.80) and a volume similarity of 0.88 (0.79–0.96). Figure 3 presents the results of a comparison of ML-estimated penumbra segmentation with the corresponding PDM-defined penumbra segmentation for two test rats. In the suture-occlusion model, at 1.5 h, the penumbra was relatively small (even sparse) in areas at the margin of a large IC. Notably, reduced DSC (0.52) and volume similarity (0.73) were observed in the rat with a relatively small extent of the penumbra, suggesting that DSC is highly sensitive to geometric changes and only considers the overlap.

**Table 4:** Evaluation metrics for penumbra segmentation using the stacking model

Penumbra	Dice similarity coefficient	Volume similarity
0.5 h	0.68 (0.48, 0.85)	0.89 (0.80, 0.97)
1.5 h	0.49 (0.31, 0.75)	0.88 (0.77, 0.96)
Overall	0.61 (0.40, 0.80)	0.88 (0.79, 0.96)

Data are presented as median and interquartile range value

**Fig. 4:**
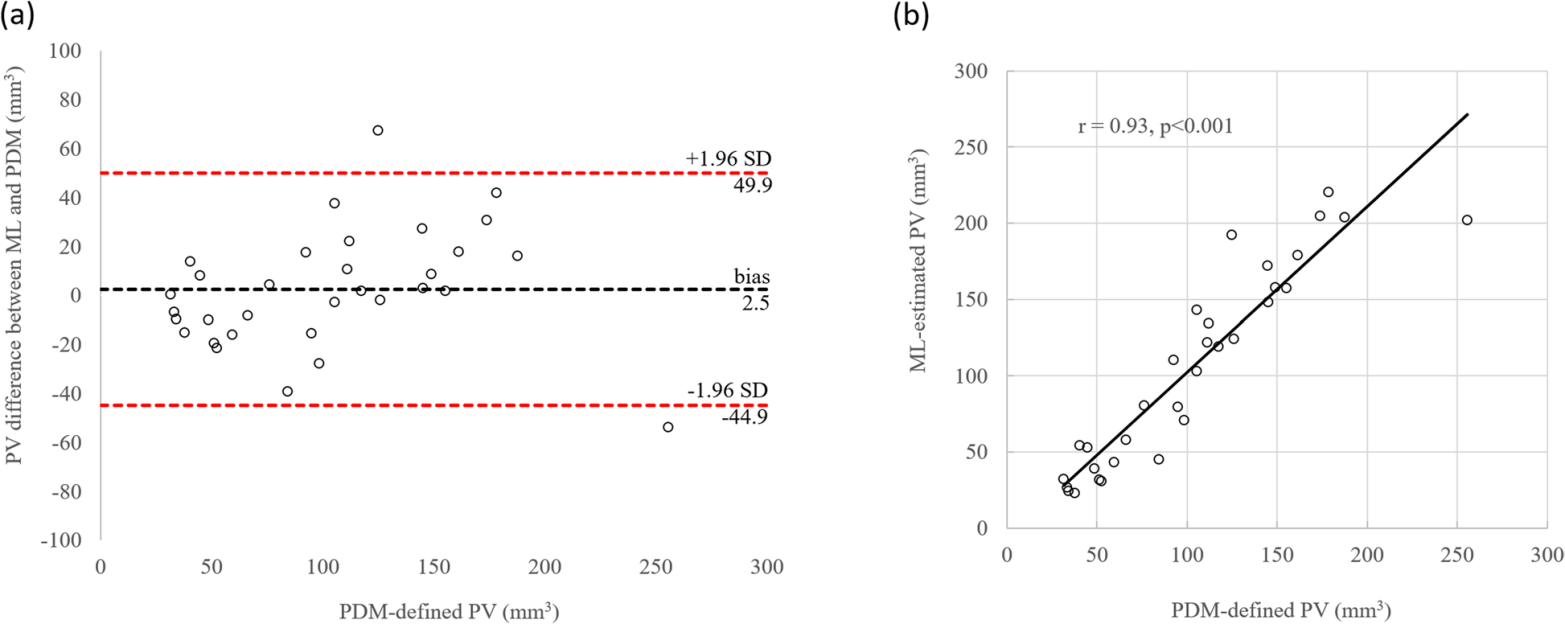
Agreement and correlations between the ML-estimated PV and the PDM-defined PV. **a** Bland*–*Altman analysis. **b** Pearson correlation analysis. *ML* Machine learning, *PDM* Perfusion–diffusion mismatch, *PV* Penumbral volume

An excellent agreement was noted between the ML-estimated PV and PDM-defined PV (Fig. 4), as indicated by high Pearson correlation coefficient (*r* = 0.93; *p* < 0.001) and the results of Bland–Altman analysis. The ML algorithm resulted in the minimal overestimation of the PV, reflected by a small positive bias (2.5 mL; 2.4% of the mean PDM-defined PV) with 95% limits of agreement ranging from -44.9 to 49.9 mL.

**Fig. 5:**
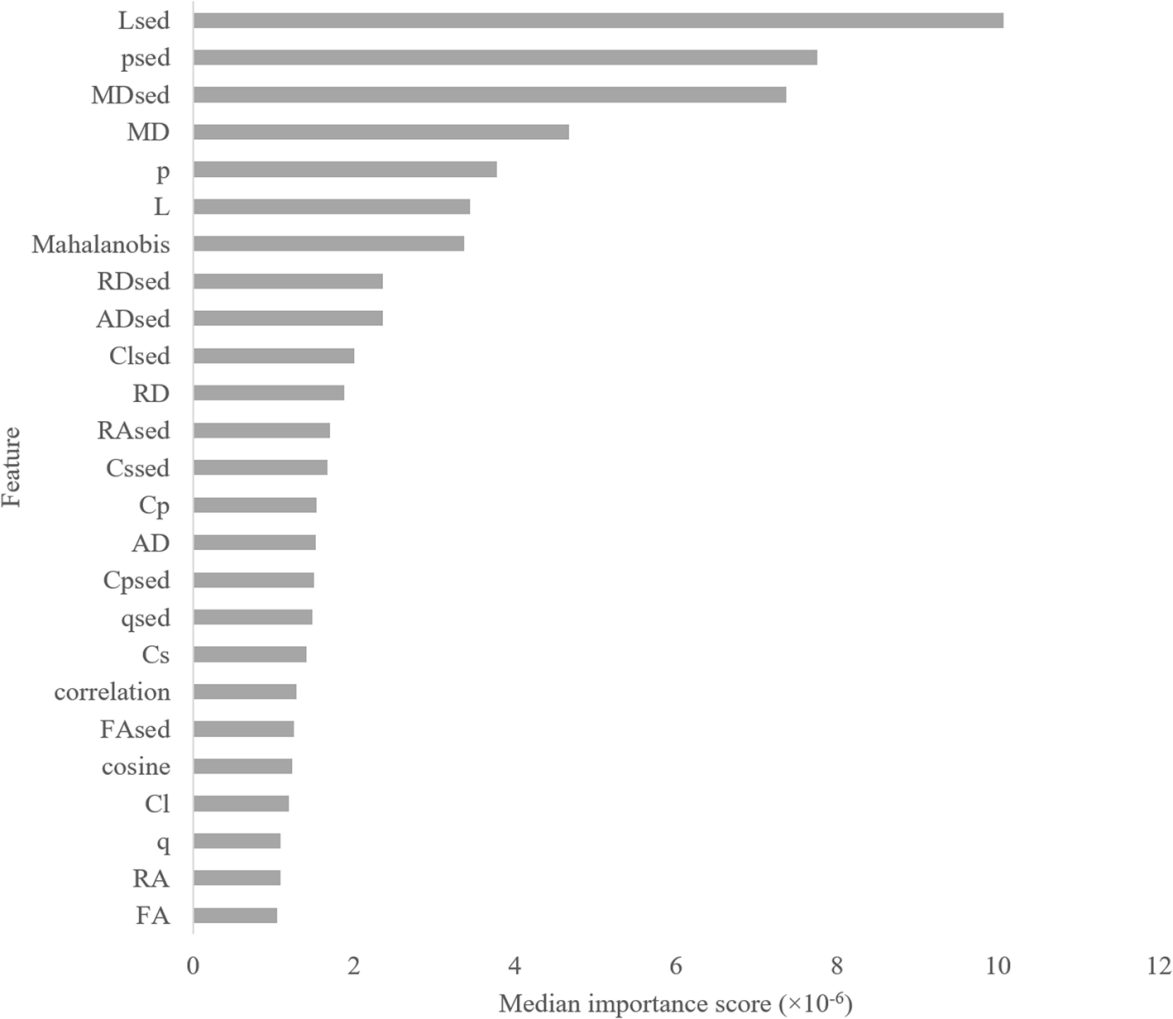
Feature importance of the random forest model. The “sed” after a feature indicates that the Euclidean distance for that feature is reported. For Mahalanobis, cosine, and correlation, their respective Euclidean distances are reported. *AD* Axial diffusivity, *Cl* Linear tensor, *Cp* Planar tensor, *Cs* Spherical tensor, *FA* Fractional anisotropy, *L* Total magnitude of diffusion tensor, *MD* Mean diffusivity, *p* Pure isotropic diffusion, *q* Pure anisotropic diffusion, *RA* Relative anisotropy, *RD* Radial diffusivity

The importance analysis of the 25 features indicated L_sed_, p_sed_, MD_sed_, MD, p, and L were the top six features (Fig. 5). These features, extracted from the penumbra class, had lower values, with a median MD of 533 × 10^-6^ (IQR 498–602) mm^2^/s, *p* of 923 × 10^-6^ (862–1,042) mm^2^/s, and L of 964 × 10^-6^ (893–1,098) mm^2^/s, than did those extracted from the NT class, with a median MD of 642 × 10^-6^ (575–699) mm^2^/s, *p* of 1,113 × 10^-6^ (996–1,211) mm^2^/s, and L of 1,157 × 10^-6^ (1,043–1,263) mm^2^/s. The values of MD, p, and L in the penumbra class were approximately 17% lower than those in the NT class (Table 5).

**Table 5:** Top six important features of the random forest model for predicting penumbra

Feature	Penumbra	NT	Difference (%)	*p*-value
L_sed_	1.00 (0.68, 1.92)	2.3 (1.6, 2.9)	-57.1%	< 0.001
p_sed_	1.11 (0.78, 2.00)	2.5 (1.7, 3.1)	-55.9%	< 0.001
MD_sed_	1.11 (0.78, 2.00)	2.5 (1.7, 3.1)	-55.9%	< 0.001
MD (× 10^-6^ mm^2^/s)	533 (498, 602)	642 (575, 699)	-17.0%	< 0.001
p (× 10^-6^ mm^2^/s)	923 (862, 1,042)	1113 (996, 1,211)	-17.0%	< 0.001
L (× 10^-6^ mm^2^/s)	964 (893, 1,098)	1157 (1,043, 1,263)	-16.6%	< 0.001

Data are presented as median and interquartile range value. L_sed_ refers to the standardized Euclidean distance for the total magnitude of diffusion tensor (L). p_sed_ refers to the standardized Euclidean distance for pure isotropic diffusion (p). MD_sed_ refers to the standardized Euclidean distance for mean diffusivity (MD)

## Discussion

In this study, we explored the potential of using DTI metrics in combination with a stack-based ensemble ML approach to estimate the PV in an animal rat model. We found a median DSC of 0.61 and a volume similarity of 0.88. During testing, the Pearson correlation coefficient between the ML-estimated PV and the PDM-defined PV was 0.93. In addition, the Bland–Altman analysis revealed a bias of 2.4%, affirming the comparability of the ML-estimated PV to the PDM-defined PV.

Our study is different from other studies in several aspects. First, we used only 25 features that are interpretable by human experts in modeling with NCA. This approach reduced the complexity of feature engineering, simplifying data preparation and enhancing the model’s clinical applicability. Second, we harnessed the power of a stack-based ensemble technique, which not only optimized segmentation performance but also eliminated the need to select a specific ML model. Finally, the feature importance analysis highlighted the three most relevant features for penumbra segmentation, *i.e*., MD, p, and L, enhancing the trustworthiness and explainability of the ML model.

In the current clinical practice, the assessment of the penumbra in patients with AIS often necessitates the injection of contrast agents for dynamic susceptibility contrast MRI. However, PWI may not always be feasible because of patient-related factors or technical difficulties [3, 4]. Therefore, a reliable method for detecting the penumbra without PWI would be of great value. Researchers have attempted to identify surrogate markers for penumbra evaluation without PWI [3, 33–35]. Deep learning models can help identify the penumbra without the use of any contrast agents [36, 37]. Similarly, we previously reported that the combination of DTI metrics and ML can effectively identify the penumbra without the need for contrast agents [38]. In the present study, we adopted a stack-based ensemble technique, substantially increasing the Pearson correlation coefficient between the ML-estimated PV and the PDM-defined PV from 0.61 [38] to our value of 0.93.

Despite its optimal performance, this ensemble technique requires extended training time and high computational resources [39]. To resolve this problem, we adopted two strategies. First, we reframed the task as a binary classification problem, exclusively focusing on the dichotomization of the non-IC region into the penumbra and NT regions within the ipsilesional hemisphere. We deliberately excluded IC segmentation because previous studies have demonstrated high accuracy (95%) in diffusion MRI–based IC segmentation [1]. This simplification resulted in a reduction in the size of training data sets, optimizing computational efficiency. Second, we performed feature selection through NCA, ensuring that only the most influential features were used to contribute to the model’s performance, thereby minimizing unnecessary computational burden. These strategies balance the trade-off between enhancing performance through the ensemble technique and managing the concomitant training costs associated with this technique.

Computed tomography perfusion (CTP) is another major technique extensively used to assess cerebral perfusion. Compared with PWI, CTP offers advantages in terms of speed and accessibility, particularly in emergency room settings. Thus, CTP has the potential to become a routine examination for patients with AIS. With the use of automated perfusion postprocessing software such as RAPID, the acquired CTP raw data can help delineate the hypoperfused and IC regions under specific thresholds. However, the arterial input function (AIF) needs to be determined to quantify perfusion [4]. Occasionally, suboptimal AIF determination may result from patient motion, misplaced AIF, low contrast bolus volume, slow injection rate, inadequate intravenous access, low cardiac output, or severe proximal arterial stenosis leading to inaccurate perfusion maps [40]. A study reported that AIF placements distal to an occluded vessel yielded inaccurate perfusion maps, whereas ipsilateral and proximal placements to the vascular occlusion produced reliable results [41]. However, despite these challenges, most patients can still benefit from treatment decision-making based on CTP-derived information. Our proposed method offers a potential alternative for cases where uncertainty or failure in perfusion map interpretation persists after CTP.

Previous studies have revealed that IC regions identified through CTP and diffusion MRI often exhibit discordance [42, 43]. In terms of accurately delineating IC regions, MRI outperforms CTP. Our ML model can not only evaluate penumbral regions without the administration of contrast agents but also concurrently provide precise delineation of IC regions on the MD map, all within a single DTI sequence. Moreover, clinicians using this model need not be concerned regarding ionizing radiation or the maximum allowable contrast dose while scanning repeatedly because of unacceptable patient movement. The proposed ML model is applicable to all patients with AIS, including older individuals, children, and pregnant women, as long as they do not have contraindications for MRI.

Computed tomography angiography is frequently used in patients with AIS to determine the occlusion site of large vessels [44], which can also be achieved through non-contrast MRI angiography such as time-of-flight imaging [45]. In summary, a comprehensive assessment at the treatment decision-making level for patients with AIS, which typically requires two doses of the contrast agent for CTP and computed tomography angiography, can be achieved using an entirely noninvasive MRI protocol including DTI and time-of-flight imaging. Although mechanical thrombectomy based on MRI findings has become a popular alternative due to the multimodal MRI protocol [46], urgent MRI access is often limited, and contraindications such as uncharacterized metallic foreign bodies can create challenges in emergency settings.

In contrast to a previous study that used only a single ML algorithm [38], our study used a stack-based ensemble framework. Although the results of McNemar test indicated that the stacking model may not consistently outperform a base model because various factors influence the success of ensemble models [47], the major advantage of ensemble methods lies in their stability, which can substantially enhance performance and reduce bias compared with single model-based approaches [48]. Moreover, the results of McNemar test demonstrated the superiority of a heterogeneous ensemble method over homogeneous ensemble methods, such as RF and boosting models; this finding is in line with those of a previous study [49].

In the medical field, the explainability of a model is vital for its clinical use because it helps medical practitioners trust ML-assisted clinical decisions. Explainability can be enhanced by incorporating features that are easily interpretable by human experts and selecting ML models with inherently high explainability [50]. However, the stack-based ensemble technique introduces an additional layer of complexity to the model, potentially making its decision-making process less transparent and comprehensible [13]. Researchers are actively exploring methods to enhance the explainability of stacking models and to make them transparent for real-world applications [51–54]. In our study, the feature permutation technique was used for the RF model [31]. Feature importance in RF is defined as the number of times a feature is selected for splitting in a node [55]. This analysis revealed that the most crucial features for penumbra segmentation were MD, followed by p and L, and the values of these three features in the penumbra class were approximately 17% lower than those in the NT class. Although a pronounced decrease in the apparent diffusion coefficient (ADC) is evident in the IC, more subtle ADC changes may remain invisible in the penumbra. Several animal [8, 10] and human [56–58] studies have revealed early minor to moderate ADC reductions in the penumbra during AIS. Our ML model can help detect the subtle ADC reduction in the penumbra that may be imperceptible during AIS, providing valuable insights into ischemic tissue injury through DTI metrics. Furthermore, we observed that FA was the least relevant feature, with no significant difference in values observed between the penumbra and NT classes (median 0.298 *versus* 0.292, respectively; *p* = 0.255). Despite FA being extensively examined as a potential diagnostic biomarker among DTI-derived metrics, its performance in the hyperacute phase remains controversial because of its definition as a ratio of q to L [59, 60]. The increase, reduction, or no change in FA values is dependent on the simultaneous analysis of q and L. However, studies have consistently reported decreased MD values [8, 38, 60], which is reflected in our importance permutation results. MD, p, and L reflect the magnitudes of molecular motion of water, which changes and becomes detectable within minutes after stroke onset. Moreover, they do not depend directly on the integrity of myelinated fiber tracts. This information on feature importance not only enhances our understanding of the model’s performance in tissue segmentation but also aligns with previous findings on temporal changes in DTI-derived metrics [8, 59].

Our study has several methodological limitations. First, the proposed ML model heavily relies on DTI metrics. However, anesthetic drugs, such as isoflurane, may inadvertently affect diffusivity [61]. Moreover, prolonged periods of anesthesia may exaggerate cell damage, making it appear more severe than it would be without anesthesia [62]. A study has described *in vivo* 7-T MRI measurements for awake animals [63]. However, it is virtually unavoidable to use anesthesia in the majority of animal stroke models. Second, the effects of gadobutrol on DTI data remain debatable. A study proposed that gadobutrol affects the measurement of eigenvalues, thereby affecting DTI data [64]. However, this study was conducted in humans, and whether gadobutrol affects DTI data in rats remains unknown. Changing the order of the imaging protocol (*e.g.*, DTI before PWI) or using the arterial spin labeling technique [36] may help circumvent potential gadobutrol interference. Finally, we used relative CBF as the threshold for defining the penumbra, whereas Tmax is widely accepted as the threshold for penumbra measurement in patients with AIS [1]. The use of different thresholds for evaluating perfusion abnormalities can lead to variations in the penumbra region, potentially affecting the ground truth (*i.e*., PDM) and subsequent model evaluation. Tmax is derived from the residual function, deconvolved by the AIF. Selecting a proper AIF for small animals such as rats remains a challenging task because of partial volume effects stemming from smaller artery diameters. Future clinical translational studies are warranted to evaluate the efficacy of our ML method in predicting PV in patients with AIS in a real-world scenario.

In conclusion, our study on an animal rat model showed the potential of an explainable DTI-based stacked model in differentiating between the penumbra and NT regions in an experimental stroke model. The proposed approach can be beneficial for patients with kidney dysfunction; it can serve as an alternative if perfusion map interpretation fails in the clinical setting.

### Supplementary Information

**Supplementary Material 1.**

## Data Availability

The datasets are available from the corresponding author on reasonable request.

## Abbreviations

ADC: Apparent diffusion coefficient
AIF: Arterial input function
AIS: Acute ischemic stroke
AUROC: Area under the receiver operating characteristic curve
CBF: Cerebral blood flow
CTP: Computed tomography perfusion
DSC: Dice similarity coefficient
DTI: Diffusion tensor imaging
DWI: Diffusion-weighted imaging
FA: Fractional anisotropy
GAM: Generalized additive model
IC: Ischemic core
IQR: Interquartile range
MD: Mean diffusivity
ML: Machine learning
MLP: Multilayer perceptron
MRI: Magnetic resonance imaging
NCA: Neighborhood component analysis
NT: Normal tissue
PDM: Perfusion–diffusion mismatch
PV: Penumbral volume
PWI: Perfusion-weighted imaging
RF: Random forest
