# Supplementary material for: Estimating the volume of penumbra in rodents using DTI and stack-based ensemble machine learning framework
Source: Eur Radiol Exp. 2024 May 15;8:59. doi: 10.1186/s41747-024-00455-z (PMC11093947; doi:10.1186/s41747-024-00455-z)
Supplement: Supplementary file 1 — Supplementary Material 1. [file 41747_2024_455_MOESM1_ESM.pdf]

Estimating the volume of penumbra in rodents using DTI and stack-based ensemble machine learning framework

ELECTRONIC SUPPLEMENTARY MATERIAL

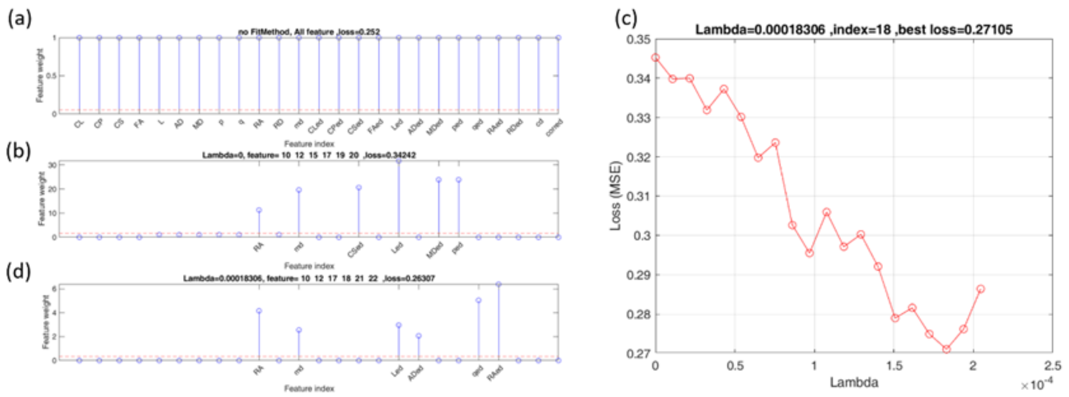

Figure S1. Demonstration of feature selection (for training) through neighborhood component analysis.

(**a**) Generalization error in neighborhood component analysis without fitting: 0.25200. (**b**) Feature weights without regularization ($\lambda = 0$); the loss value was 0.34242. (**c**) Graph of tuning regularization parameter λ; the best value of λ was 0.00018306, which resulted in the minimum mean squared error loss. (**d**) Graph depicting feature weights with $\lambda = 0.00018306$. Six features with weights of >2% of the maximum weight (red dotted line) were selected, and the loss value was 0.26307. No improvements in generalization error were observed in (**b**) and (**d**) compared with (**a**), suggesting that feature selection was not necessary in this context.

Table S1. Generalization errors in NCA without fitting, NCA fitting without regularization, and NCA fitting with regularization

Training	NCA without fitting	NCA fitting without regularization	NCA fitting with regularization
1	**0.25533**	0.34829	0.33842
2	**0.25227**	0.36469	0.25798
3	**0.26637**	0.34196	0.27438
4	**0.25750**	0.35438	0.28040
5	**0.25200**	0.34242	0.26307
6	**0.26082**	0.34349	0.27226
7	**0.26155**	0.34890	0.29062
8	**0.25735**	0.36135	0.28148
9	**0.26279**	0.35857	0.26587
10	**0.25197**	0.33820	0.34372
11	**0.25307**	0.35014	0.28559
12	**0.25124**	0.34009	0.25559
13	**0.25409**	0.34598	0.30132
14	**0.26054**	0.34894	0.26547
15	**0.25271**	0.33749	0.27064
16	**0.24614**	0.32913	0.34237

Results in bold indicate the minimum value of the generalization error in the training step. NCA, neighborhood component analysis.
